# Gene expression-based machine learning model for diagnosis, prognosis, and treatment response prediction in hepatocellular carcinoma: a retrospective study

**DOI:** 10.12701/jyms.2026.43.21

**Published:** 2026-03-04

**Authors:** Tan Thinh Nguyen, Thanh Dat Nguyen, Phu Qui Le Nguyen, Phuong Thi Bui, Minh Nam Nguyen

**Affiliations:** 1 Faculty of Medicine, University of Health Sciences, Thu Duc District, Ho Chi Minh City, Vietnam; 2 Vietnam National University Ho Chi Minh City, Thu Duc District, Ho Chi Minh City, Vietnam; 3 Research Center for Genetics and Reproductive Health (CGRH), University of Health Sciences, Thu Duc District, Ho Chi Minh City, Vietnam; 4 Faculty of Pharmacy, University of Health Sciences, Thu Duc District, Ho Chi Minh City, Vietnam

**Keywords:** Early diagnosis, Gene expression profiling, Hepatocellular carcinoma, Machine learning, Prognosis, Treatment outcome

## Abstract

**Background:**

Hepatocellular carcinoma (HCC) remains a leading cause of cancer-related mortality worldwide, largely because of challenges in early diagnosis and the limited sensitivity of conventional biomarkers. Therefore, reliable molecular tools for early detection, prognostic stratification, and individualized treatment predictions are urgently required.

**Methods:**

This retrospective study analyzed publicly available gene expression datasets. Candidate biomarkers were identified from the GSE14520 cohort using a multistep screening workflow that integrated differential expression analysis, diagnostic performance, and prognostic relevance. A 10-gene diagnostic model was constructed using least absolute shrinkage and selection operator logistic regression and subsequently validated across multiple independent cohorts. Survival outcomes were evaluated using the Kaplan-Meier analysis and treatment responses to sorafenib and transarterial chemoembolization (TACE) were assessed using receiver operating characteristic analysis.

**Results:**

A 10-gene signature (*TOP2A*, *CDK1*, *CYP3A4*, *MASP2*, *EPHX2*, *HAO1*, *RACGAP1*, *GLYAT*, *ADH1B*, and *CYP4A11*) was established. The model demonstrated robust internal performance and consistent accuracy across external validation cohorts (area under the curve [AUC], >0.9). This signature effectively identified early-stage HCC and distinguished malignancy from cirrhosis. High-risk scores were significantly associated with poor overall survival and recurrence-free survival (*p*<0.05). Furthermore, the model could predict treatment sensitivity, with higher risk scores associated with better outcomes for sorafenib (AUC, 0.791), whereas lower risk scores correlated with an improved response to TACE (AUC, 0.768).

**Conclusion:**

Our gene expression-based machine learning model provides a robust tool for HCC diagnosis, prognosis, and treatment response prediction, with potential as a supportive system for personalized clinical decision-making.

## Introduction

According to GLOBOCAN 2022, liver cancer ranked sixth in terms of incidence and third in terms of mortality among all cancers worldwide [1]. Owing to the absence of symptoms in the early-stages, most patients are diagnosed at an advanced stage, leading to a poor prognosis [2]. There is currently no established gold standard for diagnosing hepatocellular carcinoma (HCC). Although various diagnostic techniques are currently used, including computed tomography, magnetic resonance imaging, and the alpha-fetoprotein (AFP) biomarker, their reliability is frequently questioned owing to their low accuracy [3]. Moreover, recently identified HCC biomarkers generally exhibit low sensitivity, which hinders effective screening. Although AFP-L3, a subtype of AFP, is considered specific to HCC, it has demonstrated inadequate sensitivity [4]. Des-γ-carboxy prothrombin (DCP) may offer higher sensitivity than AFP, yet its levels can be influenced by the patient’s nutritional status [5]. Although the GALAD score (gender, age, AFP-L3, AFP, and des-γ-carboxyprothrombin) has been introduced as an emerging algorithm to predict the likelihood of HCC in patients with chronic liver disease, its predictive performance may vary based on the demographic characteristics of different populations, potentially leading to increased false positives [6]. Consequently, there is a critical need to develop new diagnostic models that integrate potential biomarkers using advanced machine learning algorithms.

The prognosis of patients with HCC plays a pivotal role in case management, assists in decision-making for cancer treatment, and usually relies on clinical manifestations and biomarker results. For instance, the Barcelona Clinic Liver Cancer (BCLC) classification incorporates various clinical factors, including tumor extension, liver functional reserve, and overall patient health, and categorizes patients into five stages (0, A, B, C, and D), each providing estimates of patient survival time [7]. Additionally, increased AFP levels may predict poor survival and high tumor recurrence to varying degrees, depending on whether surgery was performed or declined [8]. However, the inconsistencies in AFP levels reported among studies hinder the establishment of a universal cutoff value indicative of a poor prognosis [9]. In addition to AFP, other diagnostic biomarkers, such as AFP-L3 and DCP, have shown potential for predicting patient outcomes [9]. Imaging techniques have also emerged as noninvasive prognostic tools, but their clinical applications remain controversial [10].

Multiple treatment modalities are available for patients with HCC. Liver resection is recommended in patients with preserved liver function and adequate remnant liver volume. Liver transplantation is recommended for patients with hepatic decompensation, particularly those who meet the Milan criteria. Additional therapeutic options include radiofrequency ablation, selective internal radiation therapy, hepatic arterial infusion chemotherapy, and radiation therapy. Treatment options are selected based on the clinical condition of the patient [11]. Transarterial chemoembolization (TACE) is an effective treatment for intermediate-stage HCC, particularly in patients with a limited number of unresectable multinodular lesions, lesions in inaccessible areas, or lesions that cannot be resected because of underlying conditions. In addition to being a curative treatment, TACE can be used to induce temporary arterial embolization. To evaluate the efficacy of TACE, imaging follow-up, biomarker analyses, and liver function tests are routinely performed between therapy cycles [12]. For advanced-stage unresectable HCC, options such as embolization, ablation, and systemic therapy are considered. Oral kinase inhibitors, including sorafenib, are among the first-line systemic treatments for HCC [13]. Sorafenib inhibits several serine-threonine kinases within the mitogen-activated protein kinase pathway, a crucial signaling pathway for cell proliferation and tumor-associated angiogenesis in HCC. Research has indicated that sorafenib also induces apoptosis in cancer cells [14]. However, sorafenib has numerous side effects including dermatological conditions, diarrhea, and hypertension [15].

Technological advancements in gene sequencing and bioinformatic meta-analysis have enabled the identification of novel biomarkers capable of diagnosing and predicting outcomes in various cancers. These biomarkers have the potential to improve the detection and management of oncological diseases. Artificial intelligence (AI) and machine learning algorithms are expected to become powerful tools for identifying novel cancer biomarkers. The integration of big data analytics and machine learning algorithms holds significant promise for enhancing diagnosis, prognosis, and decision-making in cancer treatment. In this context, this study aimed to bridge these advancements by combining AI-driven approaches with biomarker discovery to develop a comprehensive model with the following objectives: (1) early diagnosis of HCC, (2) prognosis assessment, and (3) prediction of treatment response.

## Methods

**Ethics statement:** This study was conducted as a retrospective analysis using only publicly available and fully de-identified datasets obtained from open-access repositories, including the Gene Expression Omnibus (GEO) and cBioPortal. No new human participants were recruited, and no biological samples were collected. According to institutional policies and international ethical guidelines, secondary analyses of public data do not require institutional review board approval or informed consent.

### 1. Data collection and preprocessing

Publicly available gene expression datasets were retrieved from the GEO repository of the National Center for Biotechnology Information (https://www.ncbi.nlm.nih.gov/geo/) and The Cancer Genome Atlas Liver Hepatocellular Carcinoma (TCGA-LIHC) cohort via the cBioPortal for Cancer Genomics (https://www.cbioportal.org/). The inclusion criteria were as follows: (1) gene expression profiles from human HCC, cirrhotic liver tissues, or adjacent nontumor liver tissues; (2) availability of sample-level clinical information such as tumor stage, cirrhosis status, AFP, and/or treatment response; and (3) datasets containing either diagnostic labels (tumor vs. nontumor or cirrhosis vs. HCC) or survival outcomes (e.g., overall survival [OS], relapse-free survival [RFS]). Studies that used cell lines, xenograft models, or had inadequate clinical annotations were excluded. Detailed information about the studied datasets is provided in Supplementary Table 1.

Gene expression matrices and clinical information from the selected datasets were extracted and processed using platform-appropriate workflows, including robust multiarray analysis normalization for Affymetrix arrays and quantile normalization for Illumina-based platforms, followed by z-score transformation. RNA sequencing (RNA-seq) expression values from TCGA were processed using log2-transformed RNA-Seq by Expectation-Maximization values, followed by z-score normalization to ensure comparability with microarray-derived features. Patients with incomplete primary outcome information such as missing tumor/nontumor labels, unknown cirrhosis status where required, or missing survival information were excluded from the respective analyses. After preprocessing and normalization, all datasets were retained for downstream diagnostic modeling, external validation, survival analyses, and treatment response evaluation.

### 2. Gene screening

The GSE14520 cohort was used as a discovery dataset to identify candidate biomarkers with strong diagnostic and prognostic relevance in HCC. The diagnostic ability of each gene was evaluated by computing the area under the receiver operating characteristic curve (AUC) to distinguish tumor from nontumor samples. Genes with AUC >0.90 were retained as highly informative diagnostic candidates. In addition, differential gene expression (DGE) analysis was conducted between tumor and adjacent nontumor liver tissues using linear modeling and empirical Bayes moderation. Genes showing statistically significant differential expression with adjusted *p*<0.05 and |log2 fold change (FC)|>1 were considered as candidates. To incorporate prognostic relevance, survival analysis was performed for each gene using OS information, and genes with an adjusted *p*<0.05, using the log-rank test, were selected as prognostically significant. Finally, the intersection of the three gene sets was defined as the preliminary candidate gene pool for downstream model development. Gene overlap visualization and intersection analysis were performed using Venny ver. 2.1 (https://bioinfogp.cnb.csic.es/tools/venny/). Finally, probe identifiers were mapped to gene symbols using the corresponding General Public License platform annotation files. The probe-level expression values were averaged to generate gene-level matrices when multiple probes corresponded to the same gene. This screening procedure was performed as a discovery step to identify biologically and clinically relevant candidate genes in the discovery GSE14520 cohort.

### 3. Model construction

**Fig. 1.**
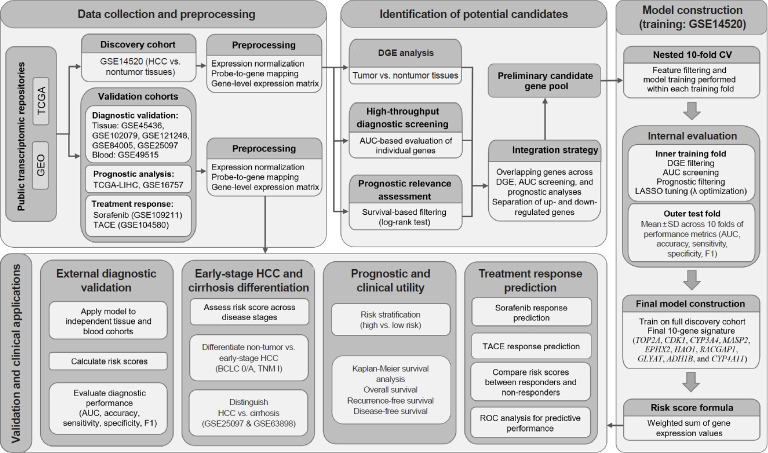
Workflow of the study. GEO, Gene Expression Omnibus; TCGA, The Cancer Genome Atlas; TCGA-LIHC, The Cancer Genome Atlas Liver Hepatocellular Carcinoma; HCC, hepatocellular carcinoma; BCLC, Barcelona Clinic Liver Cancer; DGE, differential gene expression; AUC, area under the curve; CV, cross validation; LASSO, least absolute shrinkage and selection operator; SD, standard deviation; RS, risk score; ROC, receiver operating characteristic; TACE, transarterial chemoembolization

A diagnostic model was developed using the GSE14520 cohort as the discovery dataset. A nested 10-fold cross-validation framework was implemented to ensure unbiased performance estimation and prevent information leakage. The workflow of the study design is illustrated in Fig. 1. In the outer loop, the dataset was randomly partitioned into 10 folds. For each iteration, nine folds were used as the training set and the remaining fold was reserved as an independent test set.

Within each training fold (inner loop), a complete feature-filtering procedure was performed, including DGE analysis, diagnostic AUC screening (AUC, >0.90), and prognostic filtering using log-rank tests. The resulting candidate genes were then subjected to logistic regression with least absolute shrinkage and selection operator (LASSO) regularization using the glmnet package in R. Model tuning was conducted through internal cross-validation within the training folds, with AUC specified as the optimization criterion. Subsequently, the trained model was evaluated on the corresponding outer test folds. Performance metrics including AUC, accuracy, sensitivity, specificity, and F1-score were recorded for each fold. The final internal performance was reported as mean±standard deviation (SD) across the 10 outer folds.

After completion of nested cross-validation, a final diagnostic model was constructed using the entire GSE14520 cohort. The final diagnostic model was defined using λ_min_, the value of λ yielding the highest mean cross-validated AUC. For each sample, a risk score was computed as a weighted linear combination of the gene expression values.


$$\text{ Risk score }=\text{ Intercept }+\sum_{i}\left(\beta_{i}\times {\text{ Expression }}_{i}\right)$$

where 𝛽𝑖 denotes the estimated LASSO coefficient for gene 𝑖. The optimal classification threshold was subsequently defined using the Youden index via the cutpointr package. The final 10-gene signature was derived from this full-cohort training and used for subsequent external validation analyses.

### 4. Model evaluation

Diagnostic performance of the LASSO-derived gene signature was systematically assessed across multiple independent tissue- and blood-based cohorts. Receiver operating characteristic (ROC) analysis was performed to evaluate diagnostic performance, with AUC, accuracy, sensitivity, specificity, and F1-score computed for each dataset. A series of independent external cohorts was used to evaluate diagnostic generalizability, including the tissue-based datasets GSE25097, GSE45436, GSE102079, GSE121248, GSE84005, and the blood-based dataset GSE49515.

To assess the prognostic utility of the established model, the Kaplan-Meier survival analysis was conducted in the TCGA-LIHC RNA-seq cohort and microarray datasets (GSE14520 and GSE16757) with available OS, RFS, and/or disease-free survival (DFS) information. Patients were stratified into high- and low-risk score groups using an optimal cutoff determined by the cutpointr package. Differences in survival between groups were tested using the log-rank test.

Additionally, the potential of the model to predict systemic treatment response was examined using the sorafenib-treated (GSE109211) and TACE-treated (GSE104580) cohorts. Violin plots and ROC curves were used to evaluate whether the risk score could distinguish responders from nonresponders.

### 5. Statistical analysis

Data analysis and visualization were conducted using R-Studio ver. 4.4.1 (R Foundation for Statistical Computing, Vienna, Austria) and its corresponding packages. The Wilcoxon test was used to compare differences between two sample groups, whereas the Kruskal–Wallis test was used to compare differences among more than two groups. The log-rank test was used for survival analysis. A *p*-value of <0.05 was considered statistically significant.

## Results

### 1. Identification of potential candidates

**Fig. 2.**
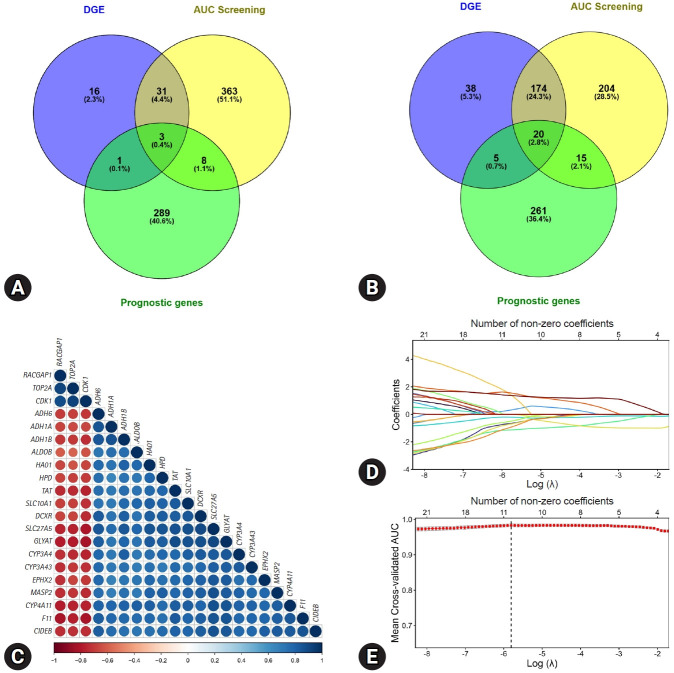
Screening and selection of candidate genes in the GSE14520 discovery cohort. (A, B) Venn diagrams depicting the overlap between differentially expressed genes, high diagnostic performance genes, and prognostic genes for upregulated and downregulated probes, respectively. (C) Correlation heatmap showing pairwise Pearson correlations among the 21 candidate genes. (D) LASSO coefficient profiles of candidate genes across a range of log (λ) values, with the number of non-zero coefficients indicated along the top axis. (E) Ten-fold cross-validation curve for LASSO logistic regression showing the mean cross-validated AUC across a range of log (λ) values. The vertical dashed line indicates the optimal λ used to determine the final diagnostic gene signature. DGEs, differentially expressed genes; AUC, area under the curve; LASSO, least absolute shrinkage and selection operator.

To systematically identify candidate biomarkers associated with HCC, we performed multistep screening using the GSE14520 discovery cohort. DGE analysis between tumor and adjacent nontumor tissues was conducted to identify significantly dysregulated probes (adjusted *p*<0.05, |log₂FC|>1). In parallel, high-throughput AUC screening was applied to evaluate the diagnostic performance of each probe by selecting probes with an AUC >0.90. Prognostic relevance was assessed using the log-rank test to identify probes that were significantly associated with OS (*p*<0.05). The intersection of DGE-significant, high AUC, and prognostically relevant probes revealed 21 candidate genes for downstream modeling (Fig. 2A, 2B; Supplementary Table 2). The Pearson correlation analysis demonstrated strong intra-signature coherence among these genes, supporting their coordinated biological behavior in HCC (Fig. 2C).

### 2. Gene selection and model construction

The 21 candidate genes identified in the discovery screening were further evaluated for diagnostic modeling using the GSE14520 cohort. To ensure an unbiased internal performance estimation and avoid information leakage, a nested 10-fold cross-validation framework was implemented. Within each outer training fold, a complete feature-filtering pipeline including DGE filtering, AUC screening, and prognostic assessment was performed exclusively on the training subset. LASSO logistic regression was then applied for feature selection and coefficient estimation, with the regularization parameter λ optimized using inner cross-validation based on the AUC.

Model performance was evaluated using the corresponding held-out test fold. This procedure was repeated across all 10 folds, and performance metrics were summarized as mean±SD to provide an unbiased estimate of diagnostic accuracy. Nested cross-validation demonstrated the high stability of the modeling framework (Table 1).

Following internal validation, the final LASSO model was trained on the entire GSE14520 cohort using the optimized λ parameter to derive the definitive 10-gene diagnostic signature (Fig. 2D, 2E). At the optimal λ_min_ value (λ_min_=0.003), 10 candidate genes (*TOP2A*, *CDK1*, *CYP3A4*, *MASP2*, *EPHX2*, *HAO1*, *RACGAP1*, *GLYAT*, *ADH1B*, and *CYP4A11*) constituted the final gene signature. These genes collectively represent both the upregulated and downregulated components of the HCC transcriptomic landscape. The risk score for each patient was computed as an intercept plus a weighted sum of gene expression levels multiplied by their respective LASSO-derived coefficients as follows:

Risk score=5.4073771+(1.3719809×*TOP2A* expression)+(0.2961392×*CDK1* expression)+

(−0.2038940×*CYP3A4* expression)+(−0.9653332×*MASP2* expression)+(−0.5160775×*EPHX2* expression)+

(1.4811425×*HAO1* expression)+(1.5722153×*RACGAP1* expression)+(−0.7314920×*GLYAT* expression)+

(−0.6738566×*ADH1B* expression)+(−1.1357714×*CYP4A11* expression)

### 3. Diagnostic performance of the established model in tissue and blood samples

The diagnostic capability of the 10-gene LASSO signature was systematically evaluated through internal nested cross-validation and independent external validation. Within the GSE14520 discovery cohort, unbiased internal performance estimated using nested 10-fold cross-validation demonstrated excellent and stable discrimination between tumor and nontumor samples, with a mean±SD AUC of 0.988±0.012. The detailed performance metrics are listed in Table 1.

To further assess generalizability, the final 10-gene model derived from the full discovery cohort was applied to multiple independent validation datasets. Across five external tissue-based cohorts (GSE25097, GSE45436, GSE102079, GSE121248, and GSE84005), the model maintained consistently high diagnostic performance, with AUC values exceeding 0.94. Notably, the GSE84005 cohort demonstrated perfect classification performance (AUC, 1.000), likely reflecting its paired tumor-adjacent tissue design, stringent pathological review, and high tumor purity. The model also performed robustly with the blood-based dataset GSE49515 (AUC, 0.910), demonstrating its potential applicability beyond tissue samples. Collectively, these findings indicate that the established model provides a highly accurate and stable diagnostic performance across diverse platforms, sample types, and clinical contexts.

### 4. Prognostic capability of the established model

**Fig. 3.**
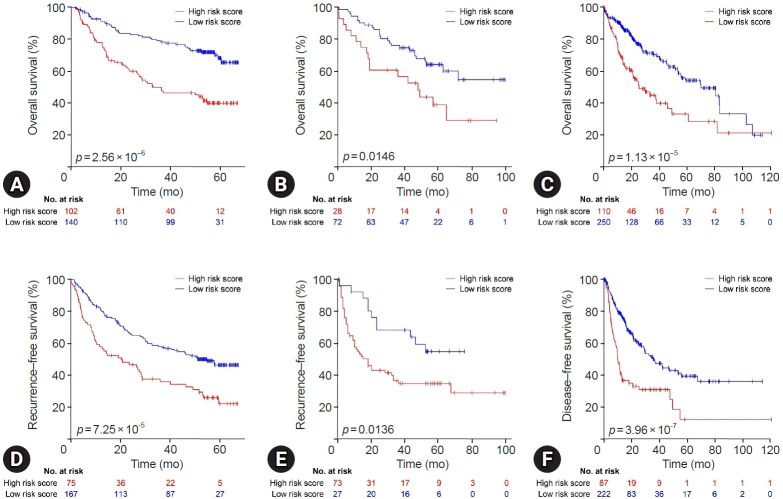
Kaplan-Meier survival analyses of the established risk model across multiple hepatocellular carcinoma cohorts. (A) Overall survival in the GSE14520 cohort. (B) Overall survival in the GSE16757 cohort. (C) Overall survival in the TCGA-LIHC cohort. (D) Recurrence-free survival in the GSE14520 cohort. (E) Recurrence-free survival in the GSE16757 cohort. (F) Disease-free survival in the TCGA-LIHC cohort. TCGA-LIHC, The Cancer Genome Atlas Liver Hepatocellular Carcinoma.

The Kaplan-Meier analyses were performed on high- and low-risk groups based on the optimal cutoff to verify whether the established risk score was associated with survival outcomes across multiple independent HCC cohorts. In the GSE14520 cohort, the high-risk group exhibited significantly poorer OS than the low-risk group (*p*=2.56×10^⁻6^; Fig. 3A). This prognostic trend was consistent in the GSE16757 cohort (*p*=0.0146; Fig. 3B), further supporting the generalizability of the risk score. When evaluated in the TCGA-LIHC RNA-seq cohort, the risk score also demonstrated significant prognostic discrimination, with patients at high risk showing markedly lower OS than individuals at low risk (*p*=1.13×10⁻⁵; Fig. 3C).

In addition to OS, the signature also predicted recurrence-related outcomes. In GSE14520, patients at high risk showed significantly shorter RFS than those at low risk (*p*=7.25×10⁻⁵; Fig. 3D). Similar results were observed for GSE16757 (*p*=0.0136; Fig. 3E), highlighting the robustness of the established model across platforms. Finally, DFS analysis in the TCGA-LIHC cohort further confirmed that the high-risk group had a substantially worse prognosis than the low-risk group (*p*=3.96×10^–7^; Fig. 3F). Collectively, these findings demonstrate that the gene signature also serves as a strong predictor of both overall and survival outcomes in HCC.

### 5. Predictive ability for transarterial chemoembolization and sorafenib treatment responses

**Fig. 4.**
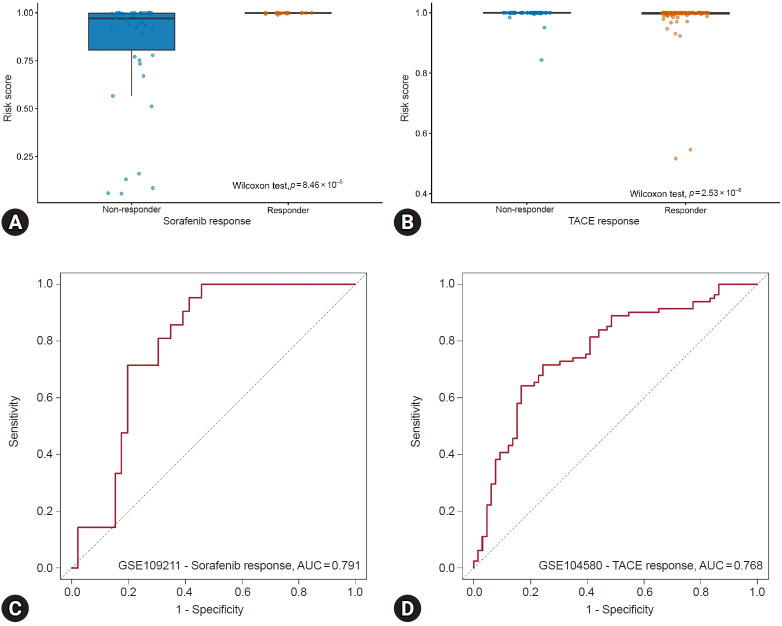
Predictive performance of the risk score for treatment responses to sorafenib and TACE therapies. (A) Distribution of risk scores between responders (n=21) and nonresponders (n=46) to sorafenib in the GSE109211 cohort. (B) Distribution of risk scores between responders (n=81) and nonresponders (n=66) to TACE therapy in the GSE104580 cohort. (C) ROC curve assessing the predictive performance of the risk score for sorafenib response. (D) ROC curve assessing the predictive performance of the risk score for TACE response. ROC, receiver operating characteristic; AUC, area under the curve; TACE, transarterial chemoembolization.

The predictive performance of the established risk score was further evaluated in treatment-response cohorts, including patients treated with sorafenib (GSE109211) and those treated with TACE (GSE104580). In the sorafenib cohort, responders exhibited significantly higher risk scores than nonresponders (Wilcoxon test, *p*=8.46×10^–5^; Fig. 4A). ROC analysis demonstrated an AUC of 0.791 (Fig. 4C), indicating a moderate predictive accuracy for distinguishing sorafenib responders from nonresponders. Conversely, in the TACE cohort, responders showed significantly lower risk scores than nonresponders (Wilcoxon test, *p*=2.53×10^–8^; Fig. 4B). The corresponding ROC curve had an AUC of 0.768 (Fig. 4D), supporting the ability of the risk score to discriminate between TACE treatment outcomes. These findings suggest that the proposed risk score not only reflects diagnostic and prognostic relevance but also holds potential as a biomarker for predicting treatment outcomes in HCC, particularly for sorafenib and TACE interventions.

### 6. Diagnostic performance in early-stage hepatocellular carcinoma and cirrhosis differentiation

**Fig. 5.**
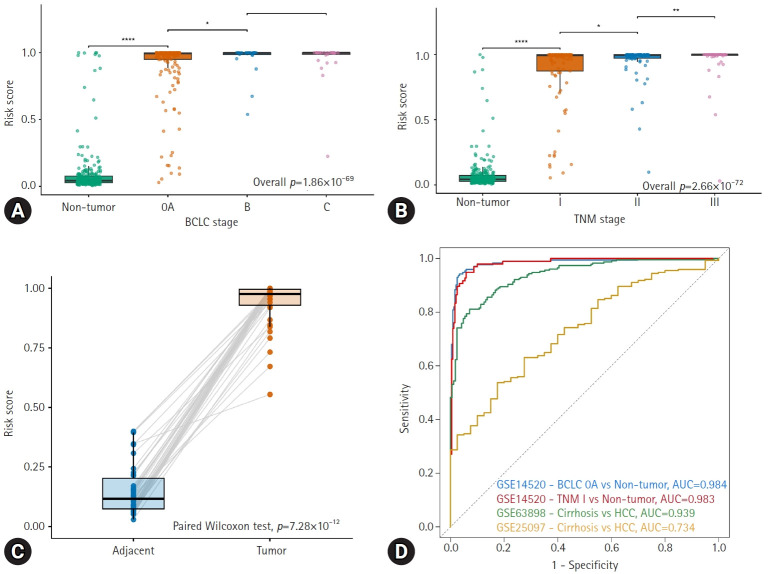
Clinical stratification and diagnostic utility of the risk score across disease stages and tissue cohorts. (A) Distribution of risk scores across BCLC stages (0/A, B, and C) in the GSE14520 cohort. (B) Distribution of risk scores across TNM stages (I, II, and III) in the GSE14520 cohort. (C) Paired comparison of risk scores between adjacent nontumor tissues and matched tumor tissues in the GSE84005 cohort. (D) ROC curves demonstrating diagnostic performance of the risk score in stage-specific and cirrhosis-versus-HCC comparisons across multiple cohorts. BCLC, Barcelona Clinic Liver Cancer; HCC, hepatocellular carcinoma; ROC, receiver operating characteristic; AUC, area under the curve. *p<0.05, **p<0.01, ***p<0.001, ****p<0.0001.

To further assess the clinical relevance of the proposed risk score, we examined its distribution across different tumor stages and its ability to distinguish early-stage HCC and cirrhosis from nontumor tissues. In the GSE14520 cohort, risk scores showed a trending stage-dependent increase from nontumor tissues to early-stage HCC (BCLC 0/A) and more advanced HCC stages (BCLC B–C), with highly significant differences across all groups (overall *p*=1.86×10^⁻69^; Fig. 5A). A similar stage-dependent pattern was observed across TNM stages I to III (overall *p*=2.66×10^⁻72^; Fig. 5B), indicating that the risk score reflects pathological advancement and tumor burden.

A paired analysis of 38 patients with HCC in the GSE84005 dataset further demonstrated the tumor specificity of the risk score. Tumor tissues had markedly higher risk scores than matched adjacent nontumor samples (paired Wilcoxon *p*=7.28×10^⁻12^; Fig. 5C), demonstrating consistent intrapatient tumor discrimination under controlled sampling conditions.

Next, we evaluated whether the risk score could effectively distinguish early-stage HCC from nontumor tissues and differentiate cirrhosis from HCC. ROC analysis showed excellent performance in distinguishing BCLC 0/A HCC from nontumor controls (AUC, 0.984) and TNM stage I HCC from nontumor tissues (AUC, 0.983) (Fig. 5D). The discriminative performance varied across cohorts for differentiating between HCC and cirrhosis. The model achieved an AUC of 0.939 in GSE63898 but demonstrated more modest discrimination in GSE25097 (AUC, 0.734).

Overall, these findings indicate that the proposed risk score retains high sensitivity for early-stage HCC detection, whereas its ability to distinguish HCC from cirrhosis may depend on cohort characteristics and underlying tissue heterogeneity.

## Discussion

Although remarkable advances have been made in HCC treatment, early diagnosis and treatment decisions remain major challenges in clinical practice. AFP, the most widely used biomarker for HCC surveillance, has been widely criticized because of its low sensitivity, particularly in the early-stage of disease progression [16,17]. Imaging-based modalities also have limitations, as early HCC and cirrhotic nodules share overlapping radiological features. Therefore, the development of molecular approaches that can enhance early detection and support clinical decision-making is warranted.

In this study, we developed a machine learning-based gene expression signature derived from a systematic multistep screening pipeline integrating diagnostic, prognostic, cirrhosis-discriminating, and treatment-response information. While the internal performance estimation provides an indication of model stability, the robustness of the proposed signature is mostly demonstrated by its good performance across multiple independent external datasets, including both tissue- and blood-based cohorts. Notably, the model maintained high accuracy across different platforms, including microarray and RNA-seq, underscoring its cross-platform technical robustness. A major clinical strength of this model is its ability to identify early-stage HCC. Specifically, the model showed excellent performance in detecting HCC at early and very early-stages (BCLC 0/A, TNM I). Early detection can improve patient survival and allow for timely, effective therapeutic interventions. Furthermore, the model showed promising yet variable results in differentiating between HCC and liver cirrhosis. While it achieved a high AUC of 0.939 in the GSE63898 cohort, its performance was more modest in the GSE25097 dataset (AUC, 0.734). This variability reflects the known histological complexity of regenerative nodules in certain cirrhotic backgrounds, which frequently mimic early malignancy [18]. It is also important to interpret the perfect classification performance observed in the GSE84005 cohort. Unlike heterogeneous clinical populations, GSE84005 comprises paired tumor and carefully curated adjacent nontumor tissues obtained from the same individuals. This paired design substantially reduces interindividual variability and maximizes molecular contrast under highly controlled pathological conditions. In this context, GSE84005 can be viewed as a proof-of-principle dataset that illustrates the intrinsic biological discriminative capacity of the proposed gene signature under optimized experimental conditions. However, this dataset should not be considered representative of typical clinical diagnostic settings, where substantial interpatient heterogeneity and diverse etiological backgrounds greatly increase complexity.

Beyond diagnosis, the model exhibited consistent prognostic value across independent cohorts. Patients at high risk had significantly lower OS, RFS, and DFS. Traditional staging systems such as BCLC and TNM, although clinically informative, cannot fully capture the molecular heterogeneity of HCC [19]. Molecular stratification can complement existing clinical techniques and improve risk-based patient counseling, particularly in intermediate-stage disease, where treatment decisions are highly individualized [20,21].

The suggested signature also exhibits potential efficacy in predicting treatment outcomes. According to the BCLC classification, TACE is the recommended first-line treatment for patients with intermediate-stage HCC. Response assessment after TACE usually depends on imaging-based criteria, such as the mRECIST (modified Response Evaluation Criteria in Solid Tumors), but these can be affected by unusual radiological patterns in some individuals [22]. Identifying molecular markers that can indicate treatment efficacy can provide more information than imaging alone. Sorafenib, a first-line systemic therapy for advanced-stage HCC, provides limited survival advantages but is often associated with various adverse effects [23]. In our study, lower risk scores were associated with improved response to TACE, whereas higher risk scores correlated with better outcomes following sorafenib therapy. The observed differential relationships indicate that the molecular profile represented by the risk signature may reflect the biological pathways associated with sensitivity to locoregional vs. systemic treatments. This information could be clinically useful for selecting the best first-line treatment and avoiding unnecessary toxicity in patients who are unlikely to benefit from specific interventions. However, owing to the exploratory nature of these analyses and the small sample size, further prospective validation is needed before they can be used in clinical practice.

Several recent bioinformatics studies have converged on mitotic regulators such as *TOP2A* and *CDK1* as recurrent hub genes in HCC, underscoring their central role in tumor biology. Ren and Feng [24] integrated three hepatitis B virus-associated HCC microarray datasets and identified 20 hub genes, among which *CDK1* and *TOP2A* were strongly associated with poor prognosis, but were not assembled into a unified diagnostic–prognostic–therapeutic model. Bao et al. [25] applied integrated differentially expressed genes, protein-protein interaction, and survival analyses in GEO/TCGA cohorts to identify overlapping hubs, such as *TOP2A* and *CDK1*, as adverse prognostic factors and potential therapeutic targets. A recent study systematically reviewed 59 studies and proposed a consensus panel of the “most representative” hub genes, repeatedly highlighting *TOP2A*, *CDK1*, *RACGAP1*, and related cell cycle genes across diagnostic, prognostic, and therapeutic contexts [26]. However, these studies stopped at gene prioritization and did not construct a single clinically deployable prediction tool. Other studies using advanced bioinformatics and drug‑repurposing strategies similarly emphasized *CDK1* and *TOP2A* as central nodes or drug targets but focused on pathway characterization or candidate compounds rather than patient‑level risk scoring [27]. Unlike prior signatures that are typically limited to prognosis or tissue-based discovery, our model is derived from a stringent screening pipeline that requires both good diagnostic performance and prognostic relevance, and is then validated across various independent cohorts with consistently high diagnostic performance (AUC, >0.9). Furthermore, by directly linking the risk score to opposite treatment‑response patterns for sorafenib and TACE, this work extends earlier hub‑gene studies from single‑task or pathway‑restricted models toward a multipurpose, clinical decision‑support tool for HCC management.

Although our gene-based model showed a robust and promising approach for HCC management, certain limitations should be considered. First, the model was validated in multiple public cohorts; however, prospective multicenter studies are required to confirm its clinical utility in real-world screening and treatment settings. Second, z-score normalization is useful for retrospective cross-platform data but may not be useful for immediate clinical use because it depends on reference distributions. Future translational initiatives will require standardized normalization frameworks or other methodologies that are appropriate for single-sample assessments. Third, although the included genes have known biological relevance, mechanistic studies are needed to further elucidate their functional roles in tumor progression and therapeutic responses, which may also reveal novel therapeutic targets.

In conclusion, we successfully constructed a novel machine learning model that enables accurate diagnosis, prognostic stratification, and prediction of therapeutic response in HCC. This integrative molecular approach represents a promising step toward precision hepatology and may contribute to improved survival and personalized treatment strategies for patients with HCC.

## Figures and Tables

**Table 1.** Diagnostic performance of the 10-gene least absolute shrinkage and selection operator model across internal and external validation cohorts

Dataset	No. of cases/controls	AUC	Accuracy	Sensitivity	Specificity	F1
GSE14520	488 (247/241)	0.988±0.012	0.971±0.028	0.976±0.028	0.967±0.047	0.972±0.026
GSE25097	557 (268/289)	0.942	0.890	0.888	0.892	0.893
GSE45436	134 (95/39)	0.992	0.985	0.989	0.974	0.989
GSE102079	257 (152/105)	0.971	0.926	0.908	0.952	0.936
GSE121248	107 (70/37)	0.947	0.944	0.943	0.946	0.957
GSE84005	76 (38/38)	1.000	1.000	1.000	1.000	1.000
GSE49515	20 (10/10)	0.910	0.950	0.900	1.000	0.947

The GSE14520 cohort was used for model training, whereas all the other datasets were used for independent validation. Internal validation of the GSE14520 cohort was performed using a nested 10-fold cross-validation framework to ensure unbiased performance estimation. Metrics are reported as mean±standard deviation across the 10 folds. The GSE84005 cohort consisted of paired tumor and adjacent nontumor tissues, which may have contributed to the observed perfect classification performance. AUC, area under the curve; F1, F1-score.

## Supplementary materials

Supplementary Tables 1 and 2 can be found at https://doi.org/10.12701/jyms.2026.43.21.

Supplementary Table 1. Overview of publicly available datasets chosen in the study

Supplementary Table 2. Profile of 21 candidate genes in discovery phase
