# Supplementary material for: Gene expression-based machine learning model for diagnosis, prognosis, and treatment response prediction in hepatocellular carcinoma: a retrospective study
Source: J Yeungnam Med Sci. 2026 Mar 4;43:21. doi: 10.12701/jyms.2026.43.21 (PMC13107085; doi:10.12701/jyms.2026.43.21)
Supplement: Supplementary Table 1. — Overview of publicly available datasets chosen in the study [file jyms-2026-43-21-Supplementary-Table-1.pdf]

Supplementary Table 1. Overview of publicly available datasets chosen in the study

No.	Data	Sample size	Chip platform	Country, year
1	GSE14520	488	Affymetrix Human Genome U133 Plus 2.0 Array & U133A Array	USA, 2009
2	GSE25097	557	Human RSTA Affymetrix 1.0	USA, 2010
3	GSE45436	134	Affymetrix Human Genome U133 Plus 2.0 Array	Taiwan, 2013
4	GSE102079	257	Affymetrix Human Genome U133 Plus 2.0 Array	Japan, 2017
5	GSE121248	107	Affymetrix Human Genome U133 Plus 2.0 Array	Singapore, 2018
6	GSE84005	76	Affymetrix Human Exon 1.0 ST Array	China, 2019
7	GSE49515	20	Affymetrix Human Genome U133 Plus 2.0 Array	Singapore, 2013
8	GSE104580	147	Affymetrix Human Genome U133 Plus 2.0 Array	Singapore, 2017
9	GSE109211	140	Illumina HumanHT-12 WG-DASL V4.0 expression beadchip	Spain, 2018
10	GSE63898	396	Affymetrix Human Genome U219 Array	USA, 2022
11	GSE16757	100	Illumina human-6 v2.0 expression beadchip	South Korea, 2023
12	TCGA-LIHC	377	RNA-seq	Multiple countries, 2018

Sample sizes indicate the number of samples included after preprocessing and quality control.
