# Supplementary material for: Gene expression-based machine learning model for diagnosis, prognosis, and treatment response prediction in hepatocellular carcinoma: a retrospective study
Source: J Yeungnam Med Sci. 2026 Mar 4;43:21. doi: 10.12701/jyms.2026.43.21 (PMC13107085; doi:10.12701/jyms.2026.43.21)
Supplement: Supplementary Table 2. — Profile of 21 candidate genes in discovery phase [file jyms-2026-43-21-Supplementary-Table-2.pdf]

Supplementary Table 2. Profile of 21 candidate genes in discovery phase

Symbol	Gene name
ADH1A	alcohol dehydrogenase 1A (class I), alpha polypeptide
ADH1B	alcohol dehydrogenase 1B (class I), beta polypeptide
ADH6	alcohol dehydrogenase 6 (class V)
ALDOB	aldolase B, fructose-bisphosphate
CDK1	cyclin-dependent kinase 1
CIDEB	cell death-inducing DFFA-like effector B
CYP3A4	cytochrome P450, family 3, subfamily A, polypeptide 4
CYP3A43	cytochrome P450, family 3, subfamily A, polypeptide 43
CYP4A11	cytochrome P450, family 4, subfamily A, polypeptide 11
DCXR	dicarbonyl/L-xylulose reductase
EPHX2	epoxide hydrolase 2, cytoplasmic
F11	coagulation factor XI
GLYAT	glycine-N-acyltransferase
HAO1	hydroxyacid oxidase (glycolate oxidase) 1
HPD	4-hydroxyphenylpyruvate dioxygenase
MASP2	mannan-binding lectin serine peptidase 2
RACGAP1	Rac GTPase-activating protein 1
SLC10A1	solute carrier family 10 (sodium/bile acid cotransporter), member 1
SLC27A5	solute carrier family 27 (fatty acid transporter), member 5
TAT	tyrosine aminotransferase
TOP2A	topoisomerase (DNA) II alpha 170 kDa
